# Building Comprehensive Toxicity Data Libraries of Short-Chain Length PHA-Based Materials for the Development of Machine Learning-Based Predictive Tools

**DOI:** 10.3390/toxics14070586

**Published:** 2026-07-02

**Authors:** Konstantina V. Filippou, Alexandros Angelis, Nikolaos P. Sotiropoulos, Marianna I. Kotzabasaki, Haralambos Sarimveis, Chrysanthos Maraveas

**Affiliations:** 1 Department of Natural Resources and Agricultural Engineering, Agricultural University of Athens, Leof. Athinon 51, 10447 Athens, Greecenikos.sotiropoulos@aua.gr (N.P.S.); mariannakotz@aua.gr (M.I.K.); 2 School of Chemical Engineering, National Technical University of Athens, Iroon Polytechniou 9, Zografou, 15772 Athens, Greece; alexandrosangelis2@gmail.com (A.A.);

**Keywords:** Polyhydroxyalkanoates, cytotoxicity, ecotoxicity, data libraries, machine learning, polymer informatics, predictive toxicology

## Abstract

Polyhydroxyalkanoates (PHAs) have emerged as a promising alternative to conventional plastics due to their biodegradable and generally favorable biocompatible profile, allowing their application in medical fields, such as drug delivery systems and surgical implants. However, the toxicity assessment of these materials is complex, time-consuming, and costly. Currently, toxicity data for PHAs are limited, dispersed across various studies, and insufficiently reported, which hinders comparative analysis and the development of predictive models. In response to these challenges, recent developments in predictive toxicology have incorporated machine learning-based approaches to estimate toxicological endpoints while reducing the reliance on in vivo experimentation. The present study aims to construct comprehensive, standardized data libraries for the cytotoxicity and ecotoxicity of PHAs and to develop and evaluate polymer-specific machine learning models that link polymer composition to toxicological outcomes. Several computational workflows were designed for this research, with Extra Trees Classifier and Gradient Boosting Classifier being the primary predictive algorithms. Furthermore, Shapley Additive Explanations (SHAP) analysis was performed to identify the descriptors that most strongly influence the predicted cytotoxicity and ecotoxicity. The cytotoxicity model achieved a test Matthews Correlation Coefficient (MCC) of 0.678 and a balanced accuracy of 0.901, with additive type, exposure conditions and particle morphology identified as the most influential descriptors. The ecotoxicity model reached a test MCC of 0.639 and balance accuracy of 0.818 within its applicability domain, with organism- and exposure-level descriptors dominating the predictions. Polymer composition contributed comparatively little, supporting the established biocompatibility of bulk PHB and PHBV. These results, however, should be interpreted in light of the modest dataset size, experimental protocols heterogeneity, and lack of independent experimental validation.

## 1. Introduction

Polyhydroxyalkanoates (PHAs) are a promising alternative to traditional plastics due to their biodegradable profile and ability to retain desirable aspects of traditional plastics without generating negative effects on the environment [1]. PHAs are produced by microorganisms that accumulate them intracellularly in the form of granules under carbon-rich and nutrient-limited conditions [2]. They demonstrate physicochemical features comparable to those of polypropylene (PP), while remaining biodegradable under aerobic and anaerobic conditions [3]. Their properties vary depending on the composition of monomers, making them suitable alternative raw materials in different industries, such as food packaging. Additionally, PHAs are generally considered biocompatible, allowing them to be applied in medical fields including drug delivery systems and surgical implants, thereby broadening their potential applications [4].

Numerous studies have built on this safety profile and assessed the biocompatibility, toxicity, and suitability of PHAs in medical and biomedical applications. Their biocompatibility arises from the monomeric units integrated into the polymers, which also naturally arise within the human body [5]. For example, polyhydroxybutyrate (PHB) is a normal metabolite found in animals, humans, bacteria, and plants [6]. Diverse studies have also revealed that PHAs do not induce thrombogenic or antigenic reactions, even during prolonged contact with biological tissues. The Food and Drug Administration (FDA) approved a member of the PHA family, specifically P(4HB), for use in resorbable structures, further confirming its safety [7]. Moreover, PHA-based materials and blends have been shown to support cell adhesion, proliferation, and formation of tissues, making them highly attractive for biomedical applications. Most recent studies have evaluated PHA-based nanoparticle formulations as carriers for therapeutic agents in cell lines such as A549, HEK 293, and Caco-2, generally confirming that observed cytotoxicity is attributable to encapsulated active compounds rather than to the polymer matrix [8].

The accumulation of increasingly smaller particles of plastic in ecosystems is a significant concern in the environment. However, the impact of bioplastics on plants and soil remains largely unclear. The effects of bio-based microplastic poly(3-hydroxybutyrate-co-3-hydroxyvalerate) (PHBV) at 0.01%, 0.1%, 1%, and 10% on soil and the health of Zea mays plants were assessed in [9]. The findings indicated that PHBV lowered plant growth and decreased foliar levels of nitrogen in a dose-dependent way. Further, PHB nanoplastics and microplastics were reported to induce adverse effects, such as reduced growth, high inhibition, and oxidative stress responses, across multiple taxa at concentration ranges similar to those reported for conventional petroleum-derived plastics [10]. Recent studies have further reported sublethal effects of PHB and PHBV micro- and nanoparticles in aquatic invertebrates such as *Daphnia magna*, as well as in soil-dwelling organisms and crop plants [11]. As such, the development of sustainable and safe materials requires further research to evaluate their potential long-term ecological implications. However, evaluating the toxicity of chemicals, and by extension, PHAs, is complex, time-consuming, and costly [12]. It is strongly impacted by a combination of physicochemical and structural descriptors [13]. Nevertheless, potential toxic effects may not originate from the polymer itself, but from intermediate fragments, oligomers, or monomers released during biological or environmental degradation. Additional factors, such as molecular weight, the presence of additives, and the morphology of the material (e.g., membranes, fibers, and particles), further modulate biological interactions [14].

Currently, toxicity data for PHAs are limited, dispersed across various studies, and insufficiently reported, with inconsistent experimental protocols, endpoints, and exposure conditions, hindering comparative analysis or the development of predictive models [15]. Moreover, computational toxicology models are typically developed using existing datasets, such as ECOTOX [16] and ToxCast [17], which primarily contain data on small molecules. For this reason, establishing comprehensive eco/cytotoxicity data libraries for PHAs represents a pivotal effort toward understanding and mitigating potential biological and environmental risks associated with these emerging bioplastics. To the best of our knowledge, this is the first approach to incorporate experimental parameters and endpoints of cytotoxicity, acute and chronic ecotoxicity for this kind of biopolymer, in a comprehensive dataset. Importantly, the inclusion of physicochemical characteristics (e.g., monomer composition, inclusion of additives, shape, etc.) alongside biological response data facilitates the establishment of composition-toxicity relationships. Thus, this repository combines existing data to build models for predicting environmental toxicity in a way that is more precise and less expensive than traditional tools.

Predictive toxicology has originally relied on the development of quantitative structure-activity relationship (QSAR) models. Such models have been successfully applied to predict a range of toxicological effects, including acute/chronic toxicity, genotoxicity, cytotoxicity, carcinogenicity, or ecotoxicity, mainly for low molecular weight compounds, using molecular descriptors, fingerprints, and physicochemical properties as inputs [18,19]. For example, Kotzabasaki et al. developed a Nano-QSAR model for predicting the toxicological properties of superparamagnetic iron oxide nanoparticles (SPIONs), focusing on their application as magnetic resonance imaging (MRI) contrast agents for non-invasive stem cell labelling and tracking [20]. Advances in machine learning algorithms, including random forests, support vector machines, and deep neural networks, have substantially improved predictive performance in toxicology [21,22]. Nevertheless, most predictive toxicology models are designed for small molecules and overlook critical polymer-specific characteristics, such as molecular weight, compositional heterogeneity, additive content, degradation behavior, and relevant experimental conditions, which restrict their applicability to polymeric materials [23]. Unlike classical QSAR approaches, which rely on molecular structure descriptors and topological indices derived from chemical structure, the models developed in this study employ polymer-level formulation parameters and experimental conditions as predictive inputs, an approach more appropriate for capturing the complexity of polymeric material toxicity.

In this context, the scope of the present study aims to develop comprehensive, standardized data libraries covering two complementary aspects of polymer safety: cytotoxicity, focusing on in vitro mammalian cell response, and ecotoxicity, focusing on environmentally relevant species across aquatic, terrestrial, and soil compartments. These libraries include detailed information on the composition of polymers, additives, and associated toxicological responses. Unlike existing datasets, the repositories are curated to emphasize the features specific to polymers, facilitating cross-study comparisons and providing ready-to-use data for modeling. Additionally, to mitigate the limitations of current predictive toxicology models for polymers, the study aims to develop and evaluate polymer-specific machine learning models linking polymer composition to toxicological outcomes. By accounting for formulation effects and integrating cytotoxicity and ecotoxicity data, this work seeks to improve data-driven toxicity prediction frameworks for PHAs and promote safe and sustainable by design (SSbD) material development (i.e., the proactive integration of safety and sustainability criteria during the early design phase of materials and products [24].

This work makes several distinct scientific contributions: (i) the construction of the first publicly available, cytotoxicity and ecotoxicity data libraries specifically tailored to PHB/PHBV-based formulations, integrating polymer composition, additive content, and experimental parameters in a single curated datasets; (ii) the development of ML classification models that link structural descriptors and experimental conditions to toxicological outcomes, which is an approach not previously applied to this class of biopolymers; (iii) the use of SHAP analysis to identify and interpret the descriptors that most strongly drive toxicity predictions, providing mechanistic interpretations in addition to predictive accuracy; and (iv) the deployment of the trained models on the Jaqpot cloud platform, enabling open, reproducible access to the predictive tools via a standardized web interface and API.

## 2. Materials and Methods

### 2.1. Data Collection

For the cytotoxicity and ecotoxicity data libraries of short-chain length PHAs (scl-PHAs), i.e., formulations of PHB/PHBV, a systematic literature mining was conducted to collect the required data for the study. A variety of peer-reviewed research papers were selected based on targeted queries focusing on physicochemical and cyto/ecotoxicity metrics for scl-PHAs and their blends with medium-chain length PHAs (mcl-PHAs), with different additives and building blocks.

Diverse scientific databases such as Scopus and PubMed were identified to search for relevant research studies. Google Scholar was also used to broaden the search scope. After identifying the databases, the next phase involved deriving keywords to ensure that relevant articles could be identified during the research. The identified keywords included “PHBV”, “PHB”, “polyhydroxybutyrate”, “polyhydroxybutyrate-co-valerate”, “poly (3-hydroxybutyrate-co-3-hydroxyvalerate)”, “toxic”, “ecotox”, “eco-toxicity”, “cytotoxicity”, “toxicology”, and “biocompatibility”. Boolean operators AND/OR facilitate quicker access to relevant articles as they define associations between keywords in a search. The operators were used to develop search phrases such as: “PHBV” OR “PHB,” AND “toxic” OR ‘’ecotox’’ AND “lignin,” OR “orotic acid,” OR “castor oil”.

**Figure 1:**
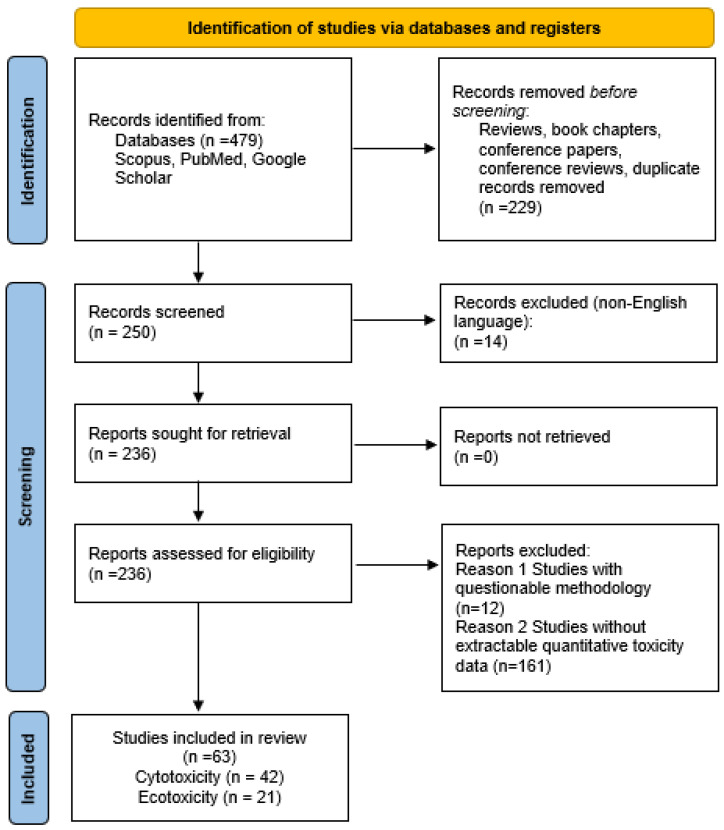
PRISMA 2020 flow diagram [25] illustrating the identification, screening, eligibility, and inclusion of studies investigating the cytotoxicity and ecotoxicity of scl-PHAs-based materials containing natural and synthetic additives.

The next phase in data collection concerned the selection of the literature. From the application of search phrases, 479 articles were identified from different scientific databases for both cytotoxicity and ecotoxicity. To select relevant studies, inclusion and exclusion criteria were defined, as shown in Table 1 below and the PRISMA 2020 flow diagram (Figure 1) [25]. The inclusion criteria emphasized studies aligned with the research topic regarding the toxicity of PHB/PHBV formulations, with or without additives, and their blends with mcl-PHAs (Table 1). Studies were limited to those that utilized primary methods for data collection and only English-published articles to avoid the need for translation. While the exclusion of non-English articles may have led to missing important insights from major agricultural countries such as Japan and China, the inclusion of only English publications was to ensure the reach to an international audience. Furthermore, English is the standard language for international publications. The focus was on full-text studies to ensure that comprehensive insights were obtained. Screening was applied in two sequential stages: a title and abstract screening pass, during which clearly off-topic records, non-research publications, and out-of-scope PHA types were excluded, followed by a full-text eligibility assessment, during which records that initially appeared eligible were evaluated against the more demanding extractability and characterization criteria. Duplicate and secondary studies were also removed, leading to 42 studies that were considered for constructing the cytotoxicity data library (Table A1 and Appendix A.1) and 21 for ecotoxicity (Table A2 and Appendix A.1).

A data extraction and curation process was performed on the most relevant research articles, resulting in the development of the cytotoxicity and ecotoxicity databases specific to PHB/PHBV formulations and their blends with mcl-PHAs. Specifically, data extraction was performed manually by a single researcher. To support quality control, the extracted data were subjected to a second-pass verification by the curating researcher, in which each entry was cross-checked against the original source publication.

For each study, relevant physicochemical, experimental, and toxicological parameters were systematically extracted by reading each article in full, capturing contextual information not always presented in structured tables within the original publications. Where quantitative data were reported exclusively in graphical form, values were digitized using PlotDigitizer, a web-based tool for extracting numerical data from plots and figures [26]. Each row in both databases represents a distinct experimental instance, where any variation at the level of material formulation or experimental setup was treated as a separate entry, preserving the original concentration- and time-dependent granularity of the data. A small number of features were derived during this database construction. In particular, polymer_percentage was calculated as the complement of the total additive content, as shown in the equation below, summing all the additives percentage in each formulation.

polymer_percentage=100−∑additives_percentages,

This calculation assumes that the polymer and its additives together constitute the complete formulation, with the polymer representing the remaining mass fraction once all the additive contributions are accounted for. All the data was systematically compiled into Comma Separated Values (CSV) files for further pre-processing.

Following the structural framework of the ECOTOX database [16], the curated ecotoxicity database included data on the ecotoxicological effects of the polymers studied at the organism, population, and ecosystem levels. Multiple endpoints were incorporated, including acute lethality (Lethal Concentration 50 (LC50), Lethal Dose 50 (LD50)), sub-lethal effects (Efficient Concentration 10 (EC10), Efficient Concentration 50 (EC50), growth inhibition, reproduction, seed germination index), developmental toxicity, and behavioral endpoints, spanning aquatic, terrestrial, and soil organisms. For data harmonization, reported endpoints were additionally mapped to toxicity categories based on the Globally Harmonized System (GHS) [27]; these classifications are reflected in the Toxicity Classification column of the ecotoxicity dataset (Table A4, Appendix A.2) and were used as the target variable for model training.

The resulting cytotoxicity database contained 440 samples described by 40 features (Table A3 and Appendix A.2), while the ecotoxicity database contained 99 samples described by 47 features (Table A4 and Appendix A.2). Both databases are publicly available via the ANIPH Zenodo repository [28,29] and include the curated raw data. The datasets were not immediately ready for modeling after curation: preprocessing steps were required (Section 2.2), including feature removal, target variable encoding, missing value imputation, outlier detection, and feature engineering.

### 2.2. Description of the Machine Learning (ML) Models

**Figure 2:**
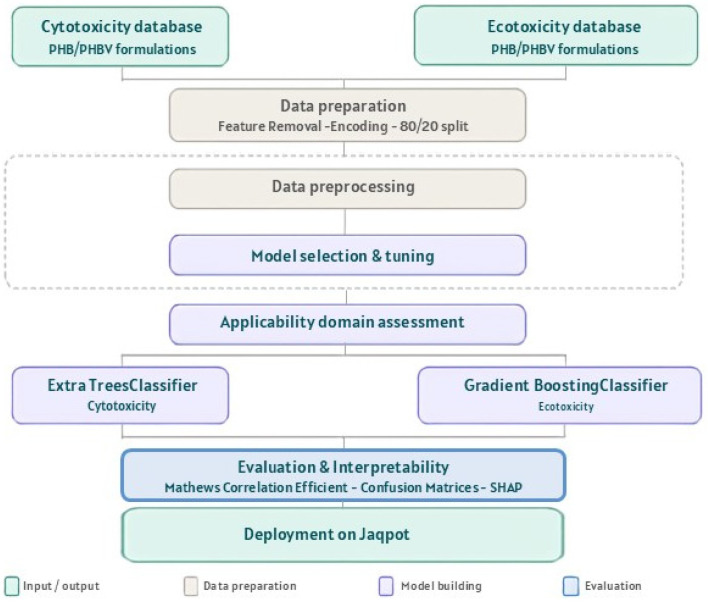
Schematic overview of the machine learning pipeline developed for the classification of scl-PHA-based material toxicity.

The development of the cytotoxicity and ecotoxicity prediction models followed the same methodological structure. Both pipelines were implemented in **Python 3.11** using key libraries such as **pandas 2.2.3**, **NumPy 2.2.5**, **scikit-learn 1.6.1**, **SHAP 0.47.2**, and **jaqpotpy7.0.x**. Each preprocessing step was fitted only on the training set and then applied to the test set, ensuring that no data leakage occurred between the two sets. An overview of the modelling pipeline is presented in Figure 1, illustrating the sequential phases from data preparation, model training, applicability domain assessment, and evaluation to final deployment on the Jaqpot platform. The sections below describe in more detail the pipeline outlined in Figure 2, explicitly noting where the two models differ.

#### 2.2.1. Initial Feature Removal

Prior to data splitting, three categories of columns were removed from each of the two datasets. The first category included columns containing identifiers (study_id, instance, etc.) as well as columns indicating the units of measurement for numerical features, such as temperature units and concentration units. The second category consists of columns that contain information directly related to the toxicity outputs of the models, such as cell viability percentage and endpoint concentrations, to avoid information leakage into the models. Finally, the third category included columns that are directly dependent on other columns, such as the percentage of monomers that sum to 100%. The exact number of columns removed differed between the databases used for the two models.

#### 2.2.2. Target Variable Encoding

For the cytotoxicity dataset, the target variable was already binary in the curated dataset, in which each experimental sample is classified as toxic or non-toxic. The binary toxic/non-toxic label in the cytotoxicity dataset is based on a 70% cell viability threshold according to the ISO 10993-5 standard [30]. For the ecotoxicity dataset, the raw target variable initially contained three classes: non-toxic, moderately toxic, and toxic. The toxic class was represented by a limited number of samples, which resulted in unstable cross-validation behavior and unreliable class-level prediction estimates. To address this, the toxic and moderately toxic classes were merged into a single toxic class, transforming the ecotoxicity task into a binary classification task consistent with the cytotoxicity modelling. This merging decision aligns with safe and sustainable by design methodology, in which, from a risk perspective, both moderately toxic and toxic categories represent materials that should not be characterized as safe [24].

#### 2.2.3. Train/Test Split

Each dataset was split into 80% training and 20% test subsets using stratified random sampling on the target label. Stratification ensures that the class distribution is maintained across both subsets, avoiding the class imbalance that is present due to the limited size of both datasets [31].

#### 2.2.4. Missing Value Handling

For both datasets, the percentage of missing values was calculated for each column on the training set only. A threshold for the percentage of missing values was then defined, above which columns were removed from both the training and test sets. For the cytotoxicity dataset, this threshold was set at 20%, apart from the concentration feature, which was retained despite its high proportion of missing values because it plays a key role as a dose-response variable. For the ecotoxicity dataset, due to the smaller training set size, a stricter threshold of 16% was applied. Following column removal, rows containing missing values were removed from both datasets. Imputation of missing values was not applied because utilizing approximated values for physicochemical features or experimental conditions could result in physically implausible inputs, which could significantly alter the formulation-toxicity dependencies that the models aim to capture. This row-removal step accounted for a substantial reduction in sample size between the raw databases and the datasets used for modelling.

#### 2.2.5. Numerical Feature Engineering

For all numerical features, the mean and standard deviation were calculated using only the training set. Features with high variability were then examined in more detail by plotting their distributions as histograms. When a feature showed multimodal patterns (distinct groups of values rather than a single continuous distribution), it was converted into categorical groups based on these observed value ranges. Additive percentage features were not converted into categories because keeping them as continuous variables provides useful information for the design of new materials. In the cytotoxicity dataset, this transformation was applied to the number of cells per well and to the concentration feature. The number of cells per well was divided into low, medium, and high categories, while concentration was divided into six categories ranging from zero to very high. No numerical feature engineering was required for the ecotoxicity dataset.

#### 2.2.6. Categorical Feature Engineering

For the ecotoxicity dataset, the media type feature consisted of a large number of categories with low frequency. These categories were grouped into four broader meaningful categories: freshwater, seawater, soil, and “other”. For both datasets, a rare-category merging step was then applied to the remaining categorical features. The frequency of each category was computed on the training set, and categories appearing in less than 5% of training samples were merged into a single “other” category. If the “other” category remained too small, it was further merged with the least frequently existing category. Finally, categorical columns that retained only a single unique category after these transformations were removed entirely. The category transformations learned from the training set were then applied to the test set to ensure that the categories in the test set matched those used during training.

#### 2.2.7. Outlier Detection

To detect outliers in the numerical features, the interquartile range (IQR) method was applied to the training set of both datasets. This method provides a robust and non-parametric approach for the detection of outliers that does not assume data normality and is less affected by skewness or imbalance in the representation of classes [32]. The IQR method is beneficial in heterogeneous and unbalanced datasets like the ones studied, where parametric alternatives, including Z-score thresholding, are likely to fail in flagging extreme values [33]. However, model training revealed that models trained without outlier removal consistently outperformed those trained with it. Therefore, no outlier removal was applied in the final pipeline of either model.

#### 2.2.8. Feature Relationship Analysis

To examine the relationships between numerical features of both datasets, Kendall’s correlation matrix was computed on the training sets. Kendall’s method was selected because it is a non-parametric measure that does not assume that data have a normal distribution and, for that reason, is more robust to the skewed distributions of both datasets. The results were plotted as a heatmap to identify highly correlated feature pairs. This process did not identify any strongly correlated feature pairs that would justify the removal of one of the variables. Therefore, no numerical features were excluded based on correlation.

#### 2.2.9. One-Hot Encoding and Scaling

As the final preprocessing step before model training, one-hot encoding was applied to the categorical features, while scaling was applied to the numerical features. Both transformations were fitted only on the training set and were then applied to the test set. This preprocessing pipeline ensures that there is no data leakage between the two sets.

#### 2.2.10. Model Development and Hyperparameter Tuning

The selection of the most suitable algorithm for the classification of toxicity in both databases was performed by comparing a series of established classifiers based on the MCC. The MCC is a reliable statistical metric that produces a score close to 1 only if the model performs well across all four possible category cases: true positives, false negatives, true negatives, and false positives. Therefore, it provides a balanced evaluation of classification performance, even when the dataset is small or the target classes are imbalanced [34]. All models were trained and evaluated using a cross-validation strategy applied to the training sets, with 10 folds for the cytotoxicity model and 5 folds for the ecotoxicity model due to the smaller number of samples in the ecotoxicity training data. The process of selecting the best classifier for each dataset was facilitated by the Pycaret library [35].

For feature selection, a recursive feature elimination procedure was applied based on the mutual information (MI) metric, which examines the dependency between each feature and the target variable using only the training set. For both datasets, the feature with the lowest MI score was removed, and the cross-validation process was repeated to assess the impact of its removal. In both cases, model performance deteriorated after feature removal. For this reason, all features were retained in the final models for both datasets.

Ultimately, the Extra Trees Classifier was chosen for the cytotoxicity dataset and the Gradient Boosting Classifier for the ecotoxicity dataset. The optimal hyperparameters for each classifier were identified using Bayesian optimization through the Optuna framework [36], with MCC again used as the optimization objective.

#### 2.2.11. Definition of the Applicability Domain

Before evaluating the performance of both models on the test sets, the applicability domain (AD) of each model was defined based on the feature space covered by the corresponding training set. The methods used to assess the AD were the leverage and bounding box methods. Samples from the test sets that were found to be outside of AD were excluded from the final evaluation of the models, ensuring that the performance of the models reflects predictions made within the feature space on which each model was trained.

#### 2.2.12. Model Evaluation

The performance of the models was evaluated based on the MCC metric described before. In the cytotoxicity model, due to the unequal distribution of classes (86 non-toxic compared to 23 toxic samples), the class weight parameter of the Extra Trees Classifier was set as balanced, which leads the algorithm to pay more attention to the underrepresented class. On the contrary, in the ecotoxicity model, due to the approximately equal distribution of classes (30 non-toxic and 29 toxic samples), no weighting was needed. Confusion matrices were also calculated for each model to visualize the results and to examine false negative predictions, particularly cases in which toxic materials are incorrectly classified as non-toxic. This type of misclassification is a direct design risk and contradicts the principles of SSbD materials.

#### 2.2.13. SHAP Analysis

To assess the interpretability of the developed models, SHAP analysis was performed. SHAP assigns an importance value to each feature for a given prediction and provides both global and local interpretability of the model’s outcomes [37]. SHAP summary plots were generated for both models on the test sets, showing the contribution of each feature to toxicity predictions on unseen data. This analysis highlighted the features that mainly drive toxicity predictions, supporting decision-making within the framework of SSbD materials.

#### 2.2.14. Model Deployment on the Jaqpot Cloud Platform

The final machine learning models were deployed on the Jaqpot platform to make the developed cytotoxicity and ecotoxicity classifiers accessible to the community through a standardized online environment. Jaqpot is a cloud-based environment designed for hosting, managing, and executing predictive models in chemistry, materials science, biotechnology, and toxicology. The platform enables users to submit new input data and obtain toxicity predictions directly from the deployed models. In addition, the models can be accessed through application programming interfaces (APIs), allowing their integration within larger workflows and decision-support systems.

## 3. Results

### 3.1. Cytotoxicity Model

#### 3.1.1. Dataset Composition and Input Features

Following the methodology analyzed in Section 2 for preprocessing and train/test splitting, the final cytotoxicity dataset consisted of 109 samples in the training set and 27 samples in the test set. The classification task was treated as a binary case where each sample was labeled as *toxic* or *non-toxic*. The total input features retained after preprocessing and a sample of 20 rows from the training set are summarized in Table 2 and Table 3, respectively.

To examine the relationship between each input feature and the toxicity target, MI scores were computed on the training set as illustrated in Table 4. The highest MI scores were recorded for *Additive1 percentage* (0.316) and *Polymer percentage* (0.302), while the lowest scores were seen for *Assay* (0.034) and *Number of cells per well* (0.023). As mentioned in Section 2, despite their MI scores, all input features were retained in the final model because feature removal did not improve cross-validation performance.

**Figure 3:**
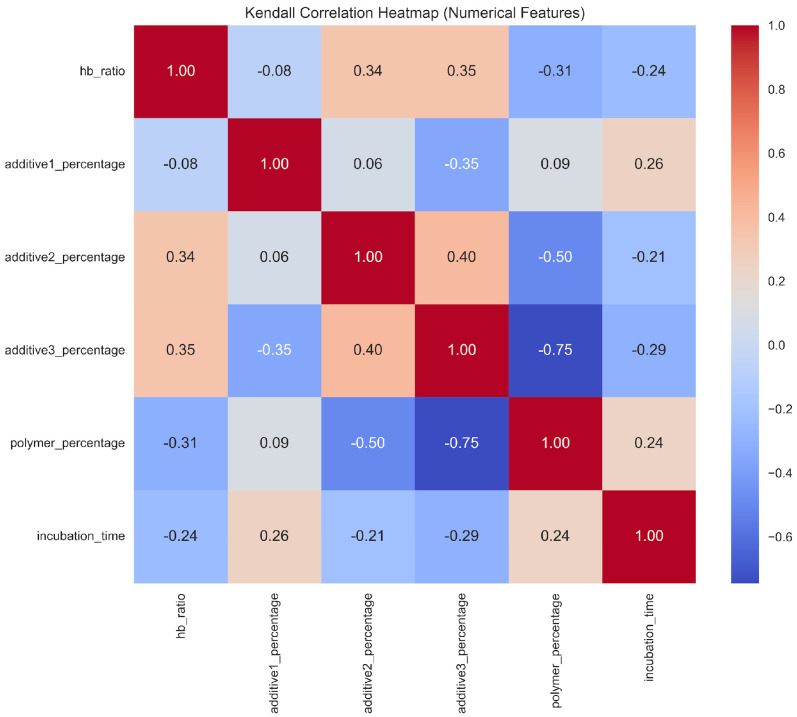
Kendall correlation matrix for input features in the training set (cytotoxicity dataset).

As far as the relationships among the numerical input features are concerned, Kendall’s correlation matrix was computed on the training set as shown in Figure 3. The strongest correlation was observed between *Additive3 percentage* and *Polymer percentage*, with a correlation coefficient of −0.75, which is expected since these fractions are compositionally linked. All other numerical features show moderate correlations with one another, suggesting that multicollinearity was not a major concern and that no numerical feature removal was justified on this basis.

#### 3.1.2. Model Selection and Hyperparameter Tuning

The algorithm selection process using PyCaret indicated that the Extra Trees classifier was the most suitable algorithm for the final cytotoxicity model. The optimal hyperparameters of the model, derived using the Optuna framework with MCC as the optimization objective and 10-fold cross-validation, are shown in Table 5.

#### 3.1.3. Model Performance

During CV evaluation, the model achieved an MCC score of 0.678 and a balanced accuracy of 0.901, indicating good generalization without signs of overfitting. Prior to model evaluation on the test set, the test samples were assessed to determine whether they fell within the AD of the model, which, as mentioned previously, was defined using the leverage and bounding box methods. A sample was considered to be within the AD only if it was classified as in-domain by both methods. Of the 27 samples in the test set, one was identified as out-of-domain and was, therefore, excluded, resulting in 26 samples for final evaluation. The evaluation of the test data resulted in an MCC score of 0.753 and a balanced accuracy of 0.925, demonstrating good predictive performance on previously unseen data.

**Figure 4:**
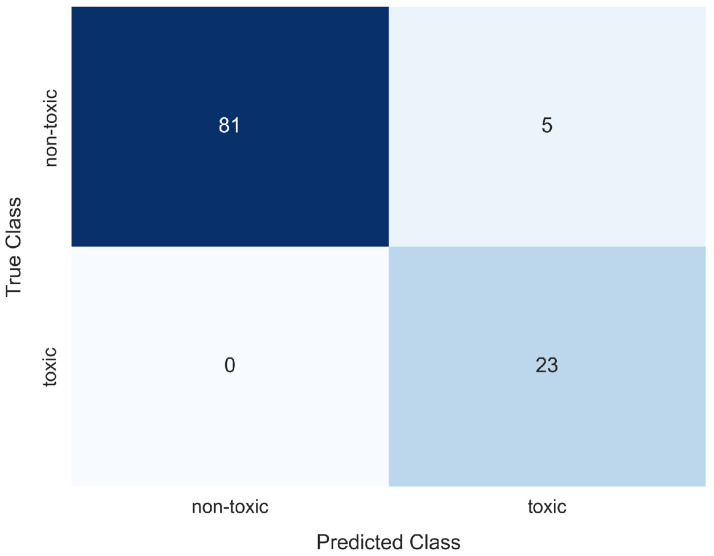
Confusion matrix for the train set (cytotoxicity model).

**Figure 5:**
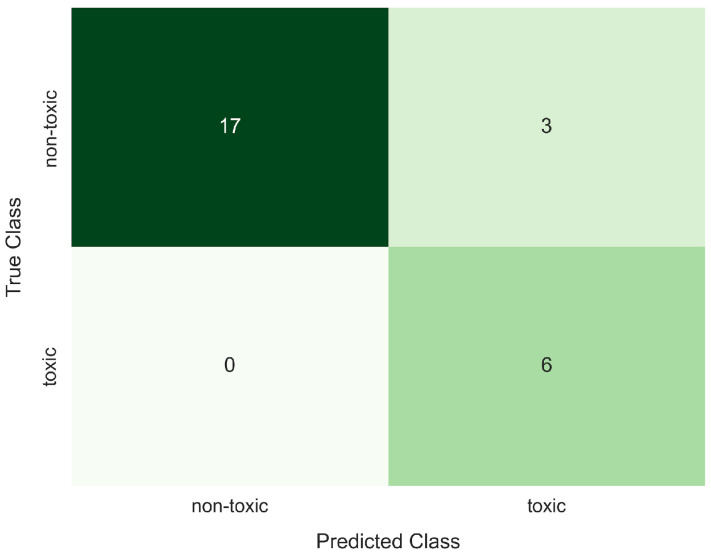
Confusion matrix for the test set (cytotoxicity model).

The confusion matrices presented in Figure 4 and Figure 5 correspond to the training and testing sets, respectively. In both cases, the good performance of the model is confirmed. Importantly, no truly toxic samples were incorrectly classified as non-toxic in either the training or test set, which would raise safety and risk concerns. However, it should be noted that the test set contains a relatively small number of toxic samples (approximately 6 out of 26 samples after AD filtering), and the absence of false negatives should be interpreted with appropriate caution.

#### 3.1.4. SHAP Explainability Analysis

**Figure 6:**
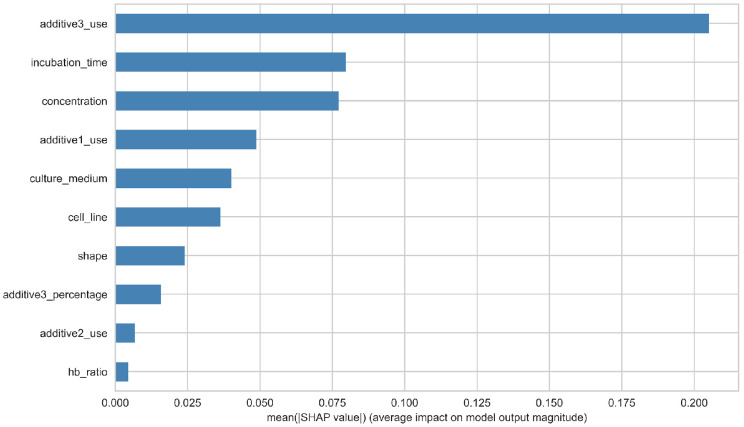
Mean absolute SHAP values of the top 10 input features of the cytotoxicity model.

**Figure 7:**
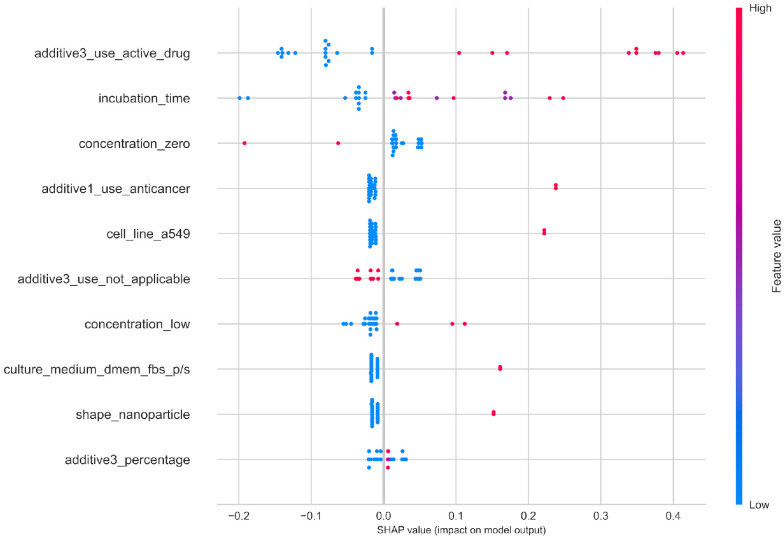
SHAP beeswarm plot illustrates the impact of the top 10 input numerical features or categories on the cytotoxicity model predictions.

SHAP analysis was performed on AD-filtered test samples to identify features that strongly impacted the predicted cytotoxicity, as demonstrated in Figure 6 and Figure 7.

The bar plot in Figure 5 revealed that *additive3_use* was the most influential feature with a mean absolute SHAP value more than twice that of the second in the ranking feature. The beeswarm plot in Figure 7 confirmed this result, identifying *additive3_use_active_drug* as the most impactful category, while *incubation time* was the most influential numerical feature. The presence of an active drug as the third additive and longer incubation times contribute to shifting model predictions towards the toxic class.

### 3.2. Ecotoxicity Model

#### 3.2.1. Dataset Composition and Input Features

The final ecotoxicity dataset consisted of 59 samples for the training set and 14 samples for the test set. The classification task was treated as a binary case where each sample was labeled as *toxic* or *non-toxic*. The total input features retained after preprocessing and a sample number of 20 rows from the training set are summarized in Table 6 and Table 7, respectively.

**Figure 8:**
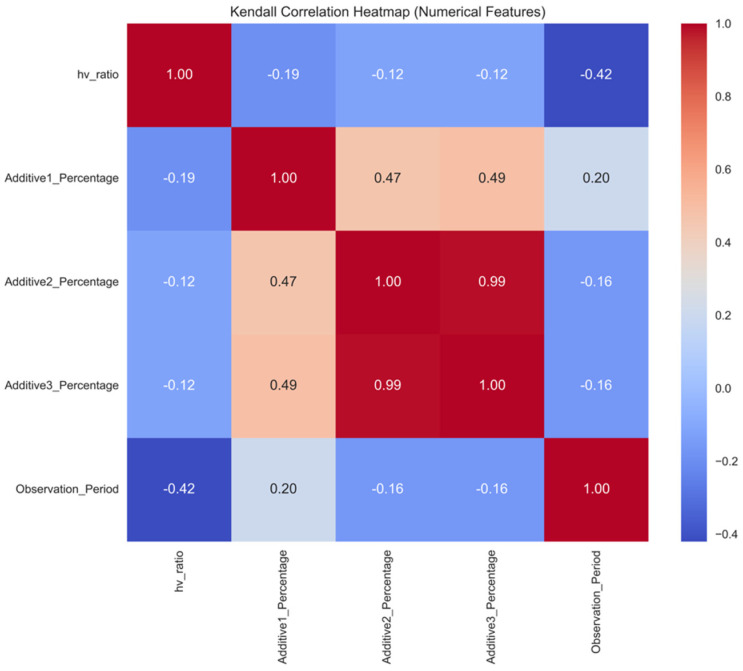
Kendall correlation matrix for input features in the training set (ecotoxicity dataset).

In accordance with the cytotoxicity model, MI scores and Kendall’s correlation matrix were computed as shown in Table 8 and Figure 8, respectively.

The highest MI scores were recorded for *Observation Period* (0.233) and *Media type* (0.203), while the lowest scores were observed for *Additive3 type* (0.061) and *hv ratio* (0.004). Despite differences in their MI scores, all input features were retained in the final model because their removal did not improve cross-validation performance. In particular, the 3HV percentage should not be removed from the dataset, as it is an important design parameter.

As far as the relationships between numerical input features are concerned, a strong correlation was observed between Additive2 Percentage and Additive3 Percentage, with a correlation coefficient of −0.99. However, this correlation reflects the specific formulations represented in the current dataset rather than a structural constraint, as evidenced by the much lower correlations observed for the same features in the cytotoxicity dataset. Since each of these features contains distinct design-relevant information, and their removal did not improve the cross-validation metrics, they were retained in the model. However, it is noted that additional experimental data would help further distinguish the individual contribution of each of these parameters to the model predictions. All other numerical features showed moderate correlations with one another, suggesting that multicollinearity was not a major concern for the remaining numerical variables.

#### 3.2.2. Model Selection and Hyperparameter Tuning

The algorithm selection process using PyCaret indicated that the Gradient Boosting Classifier was the most suitable for the final ecotoxicity model. The optimal hyperparameters of the model, identified using the Optuna framework with MCC as the optimization objective and 5-fold cross-validation, are shown in Table 9.

#### 3.2.3. Model Performance

**Figure 9:**
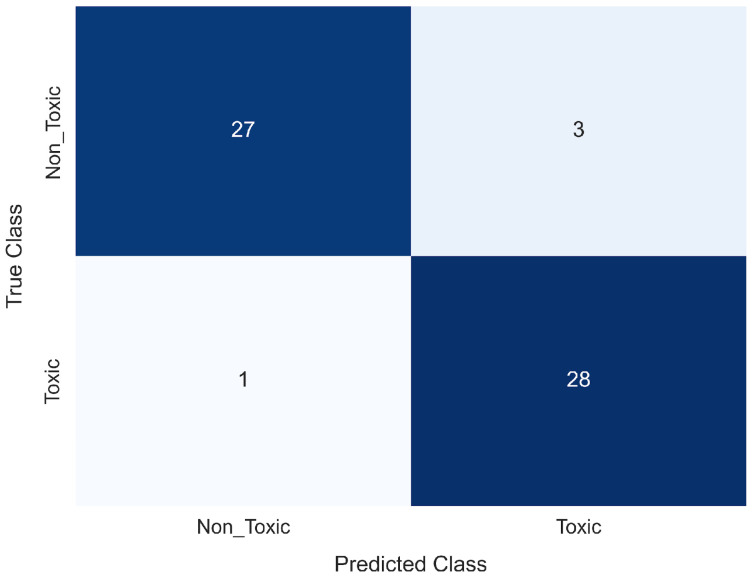
Confusion matrix for the train set (ecotoxicity model).

**Figure 10:**
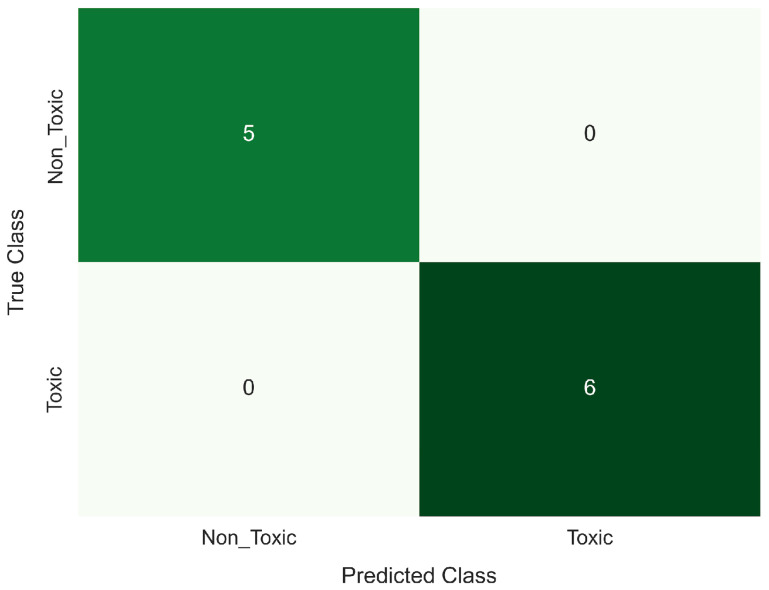
Confusion matrix for the test set (ecotoxicity model).

During cross-validation, the model achieved an MCC score of 0.639 and a balanced accuracy of 0.818, indicating good generalization, without signs of overfitting. Prior to model evaluation on the test set, the applicability domain of the ecotoxicity model was assessed. Three samples were considered out-of-domain and were, therefore, excluded, resulting in 11 samples for the final evaluation. Evaluation on the test set resulted in an MCC score of 1.0 and a balanced accuracy of 1.0, indicating perfect classification performance on the retained unseen samples. However, this result should be interpreted with caution because of the small number of samples in the final test set. The confusion matrices presented in Figure 9 and Figure 10 correspond to the training and test sets, respectively. In both cases, the results confirm the good performance of the model. It should be mentioned that in the training set, one truly toxic sample was misclassified as non-toxic (false-negative prediction).

#### 3.2.4. SHAP Explainability Analysis

**Figure 11:**
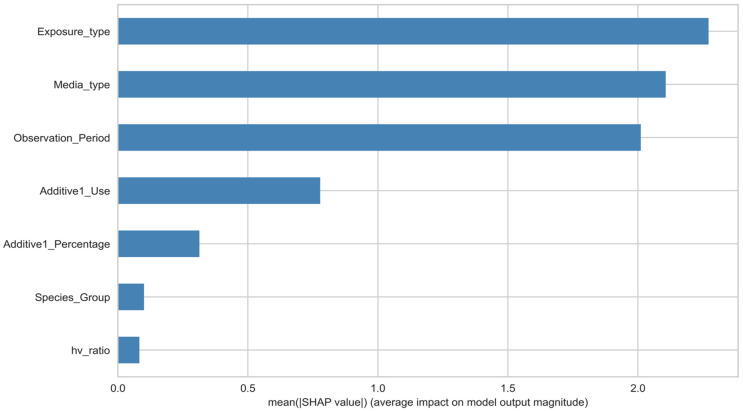
Mean absolute SHAP values of the top 7 input features of the ecotoxicity model.

**Figure 12:**
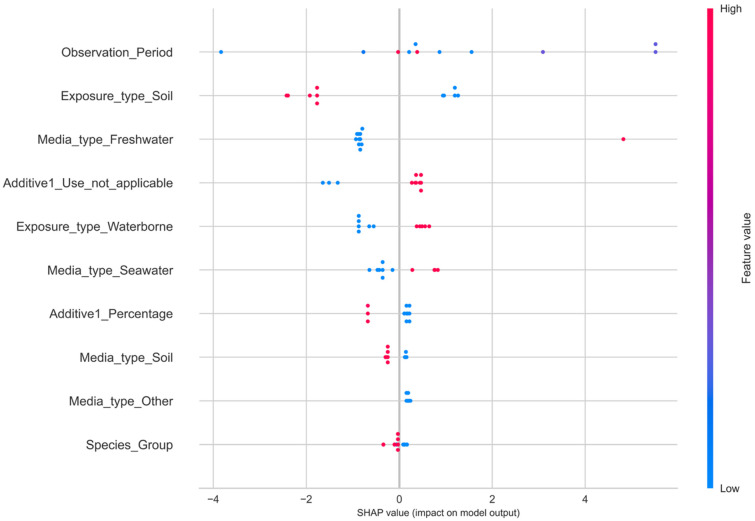
SHAP beeswarm plot illustrates the top 10 input numerical features or categories on the ecotoxicity model predictions.

SHAP analysis was performed on AD-filtered test samples to identify features that strongly impacted the predicted ecotoxicity, as demonstrated in Figure 11 and Figure 12.

The bar plot in Figure 10 revealed that *Exposure type*, *Media type,* and *Observation Period* were by far the most influential features. The beeswarm plot in Figure 11 verified these results, identifying *Observation Period* as the most influential numerical feature, while *Exposure type*—*soil* with *Media type*—*freshwater* were the most impactful categorical features. Subsequently, the SHAP results were indicative that the model primarily relied on duration of exposure and experimental conditions to distinguish between classes of ecotoxicity.

## 4. Discussion

The results of this study revealed that machine learning classification models can effectively capture patterns in the ecotoxicity and cytotoxicity datasets used for PHBV-based polymers, despite the challenges posed by the limited sample size and categorical toxicity labels. Both models achieved high MCC and balanced accuracy scores on the training and test sets, indicating satisfactory class discrimination without bias towards the majority class.

The SHAP and feature importance analysis revealed that additive percentages were among the descriptors exerting the strongest impact on the predicted ecotoxicity and cytotoxicity outcomes. For the cytotoxicity model, the dominant influence of *addtitive3_use*, driven by the *active_drug* category, reflects the inclusion of PHBV nanoparticle formulations engineered as carriers for therapeutic agents such as paclitaxel and ellipticine, whose cytotoxic effect on the tested cell lines is the intended pharmacological outcome rather than a property of the PHBV matrix. The strong contributions of *incubation_time* and *concentration* mirror the two best-established determinants of in vitro cytotoxicity outcomes, summarizing the classical dose-response pattern that systematic reviews consistently identify as dominant across nanomaterial and biopolymer studies [38]. Furthermore, the contribution of *shape_nanoparticle* is consistent with the well-documented size-dependent cellular uptake and oxidative-stress mechanisms reported for polymer micro- and nanoparticles [39]. Therefore, this observation was aligned with previous reports, which indicated that additives and plasticizers dominate the toxicological profile of polymeric systems due to potential leaching, degradation, or bioavailability in biological and environmental media [40,41].

In the ecotoxicity model, the dominance of organism- and exposure-level descriptors (species group, exposure type, observation period) reflects the multi-compartment nature of environmental exposure, where toxicity outcomes depend more strongly on the route and duration of exposure across diverse taxa than on the specific polymer grade [11].

In both cases, exposure-related parameters, like concentration and incubation or preservation or observation time demonstrated a significant predictive effect. Higher exposure concentration and longer exposure durations were linked with higher predicted toxicity, consistent with established dose-response relationships in ecotoxicology and toxicology [42]. These findings strengthen the importance of considering experimental conditions when interpreting toxicity results across different studies. Consequently, although scl-PHAs are widely recognized as biocompatible and biodegradable polymers, the integration of functional additives can significantly modify their environmental and biological behavior.

When comparing the two models, differences in the most influential parameters emerged that reflect the distinct nature of each endpoint. For the cytotoxicity model, toxicity outcomes were primarily driven by formulation-specific parameters (polymer and additive percentages). For the ecotoxicity model, organism-level descriptors (species group, exposure type) and temporal parameters (observation period) were among the dominant predictors. From a toxicological standpoint, this is expected, since exposure route and duration are key determinants of ecotoxicological outcomes.

Conversely, polymer composition, such as the monomeric ratios of 3HB and 3HV, had a relatively limited impact on the model predictions. This suggests that, within the composition ranges represented in the dataset, variation in the PHBV matrix contributed less to the predicted toxicity outcomes than additive-related and exposure-related factors. These findings support the existing literature describing PHBV and related PHAs as generally biocompatible and biodegradable materials with low intrinsic toxicity, where observed adverse effects are often related to degradation products, additives, or environmental conditions rather than the polymer itself [43].

Despite the positive results, several limitations were also identified. Given the available data volume, model development focused on conventional machine-learning algorithms rather than data-demanding architectures such as deep learning, while the limited dataset size should be considered when interpreting validation performance and model generalizability. In addition, data were integrated from multiple literature sources with different experimental protocols. Although this heterogeneity introduces variability, it also allows the models to capture a broader range of real-world experimental conditions rather than being limited to a single standardized protocol. Finally, the models were developed using curated bibliographic data and evaluated through internal validation. A stratified random train/test split was used to preserve class balance and data representativeness, while strict publication-level and formulation-level grouping was not applied because of the limited and uneven distribution of records across studies and formulations. Therefore, the models should be applied within the chemical, formulation, and experimental space covered by the current datasets, as indicated by the applicability domain assessment that accompanies model predictions.

To sum up, the results indicated that interpretable machine learning models can be effectively applied to toxicity classification for polymeric materials, provided that the results are interpreted within the bounds of the available data and without overextension toward mechanistic explanations. The combination of balanced performance metrics and explainability tools such as SHAP provides a practical framework for exploration, toxicity modeling, and hypothesis generation, especially when data are limited.

## 5. Conclusions

The current study developed and assessed machine learning-based classification models for predicting cytotoxicity and ecotoxicity of PHBV-based polymers using data derived from the literature. The use of Tree-based classifiers revealed a strong and consistent predictive performance across both endpoints, achieving high MCC and balanced accuracy scores on the independent test sets and during cross-validation.

Model interpretability analysis based on SHAP provided insights into the relative value of input features, highlighting the dominant impact of species-related descriptors, exposure conditions, and formulation parameters on toxicity predictions. While such findings reflect statistically meaningful patterns within the datasets, they should be interpreted as predictive relationships rather than direct biological mechanisms.

These findings should be interpreted within the limitations of the study, namely the modest dataset size, the heterogeneity of source experimental protocols, the absence of independent external experimental validation, and the limited statistical power of test set performance estimates after applicability domain filtering. Future expansions of the datasets and prospective experimental validation will be essential to confirm and extend the predictive scope of the developed models.

Overall, this study demonstrates the feasibility of applying interpretable machine learning models to the classification of polymer toxicity and highlights the value of structured datasets that integrate both physicochemical and experimental polymer information. The developed models provide a scientifically grounded foundation for supporting data-driven toxicity assessment of polymers, the integration of new data, future polymer structure optimization, and expansion toward broader descriptor coverage.

## Figures and Tables

**Table 1:** Inclusion and Exclusion Criteria for the Cytotoxicity and Ecotoxicity Data Libraries of PHB/PHBV Formulations.

Focus	Inclusion	Exclusion
Scope	The creation of the data libraries focused on the cytotoxicity and ecotoxicity information on PHB/PHBV formulations, with or without additives, and their blends with mcl-PHAs.	Studies not focused on cytotoxicity and ecotoxicity information on PHB/PHBV formulations, with or without additives, and their blends with mcl-PHAs.
Publication Language	English	Non-English languages, such as French, Chinese, and Spanish
Research Design	Primary Studies	Review Articles

**Table 2:** Input features used in the cytotoxicity model.

Category	Input Feature	Type	Units
Composition	3-hydroxybutyrate percentage (hb ratio)	Numerical	%
Composition	Polymer percentage	Numerical	%
Experimental properties	Concentration	Numerical	mg/L
Experimental properties	Number of cells per well	Categorical	-
Experimental properties	Incubation time	Numerical	days
Experimental properties	Shape	Categorical	-
Experimental properties	Assay	Categorical	-
Experimental properties	Cell line	Categorical	-
Experimental properties	Culture medium	Categorical	-
Additives	Additive type	Categorical	-
Additives	Additive percentage	Numerical	%

**Table 3:** Sample rows from the cytotoxicity training set.

hb Ratio	Additive1 Percentage	Additive2 Percentage	Additive3 Percentage	Polymer Percentage	No_Cells Per_Well	Concentration	Incubation_Time	Shape	Additive1 Use	Additive2 Use	Additive3 Use	Assay	Cell_Line	Culture_Medium	Toxicity
85	33.33	33.33	0	33.33	low	low	24	nanospheres	targeting_ligand	stabilizer	not_applicable	mtt	mcf7	dmem_10fbs_pen_strep	non-toxic
88	20.3	0	0	79.7	high	high	48	microsphere	filler	not_applicable	not_applicable	diic1_5	jurkat	rpmi-1640	non-toxic
88	6.6	0	0	93.4	high	very_low	24	microsphere	other	not_applicable	not_applicable	mts	hepg2	dmem_10fbs_pen_strep	non-toxic
88	12.5	0	0	87.5	high	high	48	microsphere	filler	not_applicable	not_applicable	diic1_5	jurkat	rpmi-1640	non-toxic
88	89.11	0.99	0	9.9	medium	high	24	nanoparticle	other	not_applicable	not_applicable	mtt	other	dmem_fbs_p/s	non-toxic
88	42.13	0	0	57.87	medium	medium	72	nanoparticle	anticancer	not_applicable	not_applicable	mtt	a549	dmem_fbs_p/s	toxic
88	90	0	0	10	medium	very_high	24	nanoparticle	other	not_applicable	not_applicable	mtt	other	dmem_fbs_p/s	non-toxic
88	42.13	0	0	57.87	medium	medium	48	nanoparticle	anticancer	not_applicable	not_applicable	mtt	a549	dmem_fbs_p/s	toxic
85	32.9	32.9	1.32	32.9	low	low	72	nanospheres	targeting_ligand	stabilizer	active_drug	mtt	mcf7	dmem_10fbs_pen_strep	toxic
100	33.33	33.33	0	33.33	low	very_low	72	nanospheres	targeting_ligand	stabilizer	not_applicable	mtt	mcf7	dmem_10fbs_pen_strep	non-toxic
100	24	33	32	11	medium	very_high	24	diblock_star	responsive	stealth	hydrophobe	mtt	ccd_112con	dmem_fp	non-toxic
100	32.9	32.9	1.32	32.9	low	very_low	24	nanospheres	targeting_ligand	stabilizer	active_drug	mtt	mcf7	dmem_10fbs_pen_strep	non-toxic
85	32.9	32.9	1.32	32.9	low	low	24	nanospheres	targeting_ligand	stabilizer	active_drug	mtt	mcf7	dmem_10fbs_pen_strep	toxic
100	32.9	32.9	1.32	32.9	low	low	24	nanospheres	targeting_ligand	stabilizer	active_drug	mtt	mcf7	dmem_10fbs_pen_strep	non-toxic
88	0	0	0	100	high	very_high	48	microsphere	not_applicable	not_applicable	not_applicable	diic1_5	jurkat	rpmi-1640	non-toxic
88	0	0	0	100	high	very_high	24	microsphere	not_applicable	not_applicable	not_applicable	diic1_5	other	rpmi-1640	non-toxic
88	89.11	0.99	0	9.9	medium	very_high	24	nanoparticle	other	not_applicable	not_applicable	mtt	other	dmem_fbs_p/s	toxic
85	33.33	33.33	0	33.33	low	low	48	nanospheres	targeting_ligand	stabilizer	not_applicable	mtt	mcf7	dmem_10fbs_pen_strep	non-toxic
100	23	45	8	8	medium	high	24	tetra_arm_star	responsive	stealth	hydrophobe	mtt	ccd_112con	dmem_fp	non-toxic
100	28	41	21	10	medium	medium	24	diblock_star	responsive	stealth	hydrophobe	mtt	ccd_112con	dmem_fp	non-toxic

**Table 4:** Mutual information (MI) scores for input features of training (cytotoxicity dataset).

Input Features	MI Scores
additive1_percentage	0.315861
polymer_percentage	0.301795
additive1_use	0.196738
cell_line	0.182172
additive2_percentage	0.177036
additive3_percentage	0.162405
culture_medium	0.15984
shape	0.149442
additive3_use	0.121013
hb_ratio	0.070957
additive2_use	0.063648
concentration	0.057891
incubation_time	0.038127
assay	0.033872
no_cells_per_well	0.022745

**Table 5:** Optimal hyperparameters selected for Extra Trees Classifier.

Hyperparameter	Value
n_estimators	282
max_depth	10
max_features	0.547
min_samples_split	4
min_samples_leaf	1
criterion	entropy
class_weight	balanced

**Table 6:** Input features used in the ecotoxicity model.

Category	Input Feature	Type	Units
Composition	3-hydroxyvalerate percentage (hv ratio)	Numerical	%
Experimental properties	Observation period	Numerical	days
Experimental properties	Species group	Categorical	-
Experimental properties	Exposure type	Categorical	-
Experimental properties	Media type	Categorical	
Additives	Additive type	Categorical	-
Additives	Additive percentage	Numerical	%

**Table 7:** Sample rows from the ecotoxicity training set.

hv_Ratio	Additive1_Percentage	Additive2_Percentage	Additive3_Percentage	Observation_Period	Additive1_Use	Additive2_Use	Additive3_Use	Species_Group	Exposure_Type	Media_Type	Toxicity_Classification
0	0	0	0	4	not_applicable	not_applicable	not_applicable	Aquatic_Organisms	Waterborne	Freshwater	Toxic
0	0	0	0	7	not_applicable	not_applicable	not_applicable	Aquatic_Organisms	Waterborne	Other	Non_Toxic
12	0	0	0	1	not_applicable	not_applicable	not_applicable	Aquatic_Organisms	Waterborne	Seawater	Non_Toxic
0	0	0	0	4	not_applicable	not_applicable	not_applicable	Aquatic_Organisms	Waterborne	Seawater	Toxic
0	0	0	0	4	not_applicable	not_applicable	not_applicable	Aquatic_Organisms	Waterborne	Freshwater	Toxic
0	50	0	0	28	Flexibilizer	not_applicable	not_applicable	Terrestrial_Plant	Soil	Soil	Non_Toxic
12	0	0	0	1	not_applicable	not_applicable	not_applicable	Aquatic_Organisms	Waterborne	Seawater	Non_Toxic
0	0	0	0	60	not_applicable	not_applicable	not_applicable	Aquatic_Organisms	Waterborne	Seawater	Toxic
0	0	0	0	49	not_applicable	not_applicable	not_applicable	Terrestrial_Plant	Soil	Soil	Non_Toxic
0	0	0	0	4	not_applicable	not_applicable	not_applicable	Aquatic_Organisms	Waterborne	Seawater	Toxic
0	0	0	0	1	not_applicable	not_applicable	not_applicable	Aquatic_Organisms	Waterborne	Seawater	Non_Toxic
0	0	0	0	4	not_applicable	not_applicable	not_applicable	Aquatic_Organisms	Waterborne	Other	Toxic
0	0	0	0	7	not_applicable	not_applicable	not_applicable	Aquatic_Organisms	Waterborne	Other	Non_Toxic
0	44	10	2	3	Plasticizer	Natural_Antioxidant	Chain_Extender	Terrestrial_Plant	Soil	Soil	Non_Toxic
0	0	0	0	7	not_applicable	not_applicable	not_applicable	Aquatic_Organisms	Waterborne	Other	Non_Toxic
0	0	0	0	4	not_applicable	not_applicable	not_applicable	Aquatic_Organisms	Waterborne	Freshwater	Toxic
0	0	0	0	42	not_applicable	not_applicable	not_applicable	Aquatic_Organisms	Orally	Seawater	Toxic
0	50	0	0	183	Flexibilizer	not_applicable	not_applicable	Terrestrial_Plant	Soil	Soil	Non_Toxic
6.5	0	0	0	3	not_applicable	not_applicable	not_applicable	Aquatic_Organisms	Waterborne	Seawater	Toxic
0	46.5	5	2	3	Plasticizer	Natural_Antioxidant	Chain_Extender	Terrestrial_Plant	Soil	Soil	Non_Toxic

**Table 8:** Mutual information (MI) scores for input features of training (ecotoxicity dataset).

Input Features	MI Scores
Observation_Period	0.233106
Media_type	0.203462
Additive3_Percentage	0.184351
Additive2_Percentage	0.161187
Additive1_Percentage	0.156707
Exposure_type	0.130199
Additive1_Use	0.106873
Species_Group	0.105932
Additive2_Use	0.061111
Additive3_Use	0.061111
hv_ratio	0.004256

**Table 9:** Optimal hyperparameters selected for Gradient Boosting Classifier.

Hyperparameter	Value
n_estimators	198
max_depth	2
Learning_rate	0.242
max_features	0.802
min_samples_split	5
min_samples_leaf	4
subsample	0.733
criterion	friedman_mse

## Data Availability

The datasets used for training and evaluating the machine learning models were derived from curated toxicity data reported in the literature and compiled within the framework of this study. The implementation of the ecotoxicity and cytotoxicity machine learning models, including data preprocessing, feature selection, model training, and evaluation scripts, is publicly available via GitHub at: Ecotoxicity model: https://github.com/Alexangelo92/Ecotoxicity-classification-model-for-PHBV-polymers-A.N.I.P.H..git; Cytotoxicity model: https://github.com/Alexangelo92/Cytotoxicity-classification-model-for-PHBV-polymers-A.N.I.P.H..git; In addition, the trained models and their associated feature sets are accessible through the Jaqpot platform: Ecotoxicity model: Jaqpot ID: https://app.jaqpot.org/dashboard/models/2369/description; Cytotoxicity model Jaqpot ID: https://app.jaqpot.org/dashboard/models/2367/description.

## Abbreviations

The following abbreviations are used in this manuscript:

PHA	Polyhydroxyalkanoate
PP	Polypropylene
PHB	Poly(3-hydroxybutyrate)
PHBV	Poly(3-hydroxybutyrate-co-3-hydroxyvalerate)
FDA	Food and Drug Administration
ECOTOX	ECOTOXicology Knowledgebase
QSAR	Quantitative Structure–Activity Relationship
MRI	Magnetic Resonance Imaging
SPION	Superparamagnetic Iron Oxide Nanoparticles
SSbD	Safe and Sustainable by Design
scl-PHAs	Short Chain Length Polyhydroxyalkanoates
mcl-PHAs	Medium Chain Length Polyhydroxyalkanoates
CSV	Comma-Separated Values
LC50	Lethal Concentration 50%
LD50	Lethal Dose 50%
EC10	Effective Concentration 10%
EC50	Effective concentration 50%
GHS	Globally Harmonized System
ML	Machine Learning
IQR	Interquartile range
MI	Mutual Information
MCC	Matthews Correlation Coefficient
SHAP	Shapley Additive Explanations
AD	Applicability Domain
CV	Cross-validation

## Appendix A

### Appendix A.1

**Table A1:** Studies included in the cytotoxicity data library for scl-PHA-based formulations.

Material Composition	Additive Presence	Cell Line	Endpoint	Observed Effect	Reference
PHB	No	Mammalian Cells	Cell Viability	No cytotoxic effects observed	[44]
PHBV 19% mol HV	Yes		Cell Viability		
PHBV 20% mol HV	Yes	Caco-2	Cell Viability	No cytotoxic effects observed	[45]
PHBV 2.9% mol HV	Yes	Osteoblasts	Cell Viability	No cytotoxic effects observed	[46]
PHBV 9.8% mol HV	Yes	Mammalian Cells	Cell viability	Reduced cytotoxicity observed when rifampicin was encapsulated in PHBV microparticles	[47]
PHBV 12% mol HV	Yes	A549	Cell Inhibition	Increased cytotoxic effect observed for ellipticine when delivered via PHBV nanoparticles	[48]
PHBV 6% mol HV	No	Animal Cells	Cell Viability	No cytotoxicity observed in cell viability and morphology assays using polymer extracts	[49]
PHBV 21% mol HV	Yes	L929	Toxicity Grade	No cytotoxicity observed at additive contents below 1.5 wt %	[50]
PHBV 8.6% mol HV	Yes	Mammalian Cells	Cell Viability	Cytotoxicity observed in some cases	[51]
	No				
PHB	Yes	Mammalian Cells	Number of cells/well	No cytotoxicity observed in direct and indirect contact assays	[52]
	No				
PHB	Yes	L929	Toxicity Grade	No cytotoxicity observed	[53]
PHB	Yes	L929	Cell Viability	No cytotoxicity observed	[54]
PHB	Yes	L929	Cell Viability	No cytotoxicity observed	[55]
PHBV 12% mol HV	Yes	L929	Cell Viability	No cytotoxicity observed; In some cases, cytotoxic	[56]
PHBV 5% mol HV	Yes	HEK293	Growth Inhibition percentage	No cytotoxicity observed; In some cases, cytotoxic	[57]
PHB	No	HEK	Cell Viability	No cytotoxicity observed; In some cases, cytotoxic	[58]
PHB	Yes	HaCat	Cell Viability	No cytotoxicity observed; In some cases, cytotoxic	[59]
PHBV 9.2% mol HV	Yes	Mammalian Cells	Cell Viability	No cytotoxicity observed; In some cases, cytotoxic	[60]
PHB	Yes	B16	Cell Viability	No cytotoxicity observed; In some cases, cytotoxic	[61]
PHBV 8% mol HV	Yes	SK MEL 28	Growth Constant	No cytotoxicity observed; In some cases, cytotoxic	[62]
		HepRG			
PHBV 12% mol HV	Yes	MG63	Cell viability	No cytotoxicity observed	[63]
PHB	Yes	Jurkat	Cell viability	No cytotoxicity observed	[64]
	No	HT 29	Cell viability	No cytotoxicity observed	
PHBV	Yes	Jurkat	Cell viability	No cytotoxicity observed; In some cases cytotoxic	
	No	HT 29	Cell viability	No cytotoxicity observed; In some cases cytotoxic	
PHBV	Yes	SaOS 2	Cell Viability	No cytotoxicity observed	[65]
	No				
PHBV 12% mol HV	Yes	Jurkat	Cell Viability	No cytotoxicity observed; In some cases cytotoxic	[66]
		Raji			
PHBV 6% mol HV	No	Bovine Keratocytes	Cell Viability	No cytotoxicity observed; In some cases cytotoxic	[67]
PHBV 11% mol HV	Yes	Calu 3	Cell Viability	No cytotoxicity observed	[68]
PHB	Yes	L929	Number of cells/well	No cytotoxicity observed	[69]
PHB	Yes	CCD 112CoN	Cell Viability	No cytotoxicity observed	[70]
PHB	Yes	MC3T3-E1	Cell Viability	No cytotoxicity observed	[71]
PHBV 19% mol HV	Yes	HEK293	Cell Viability	No cytotoxicity observed; In some cases cytotoxic	[72]
	No				
PHB	Yes	3T3	Cell Viability	No cytotoxicity observed	[73]
PHBV 12% mol HV	Yes	L929	Cell Viability	No cytotoxicity observed	[74]
	No				
PHB	Yes	HepG2	Cell Viability	No cytotoxicity observed; In some cases cytotoxic	[75]
PHBV 15% mol HV	Yes	MCF7	Cell Viability	No cytotoxicity observed; In some cases cytotoxic	[76]
	No				
PHB	Yes	PBMC	Cell Viability	No cytotoxicity observed	[77]
PHB	No	Chondrocytes	Cell Viability	No cytotoxicity observed; In some cases cytotoxic	[78]
PHB	No	DRG	Cell Viability	No cytotoxicity observed	[79]
PHB	Yes	HaCat	Cell Viability	No cytotoxicity observed	[80]
	No				
PHB	Yes	HepG2	Cell Viability	No cytotoxicity observed	[81]
PHBV 12% mol HV	Yes	Vero	Cell Viability	No cytotoxicity observed	[8]
	No				
PHBV 8% mol HV	Yes	Cancer cells/mouse model	Cell Viability	Reduced systemic toxicity observed in vivo for drug-loaded PHBV nanoparticles	[82]
PHBV 12% mol HV	No	SW480	Cell viability	Concentration-dependent effects observed; no cytotoxicity at low concentrations	[83]
	Yes				
PHB	No	L929	Cell viability	No cytotoxicity observed; cell viability maintained after 72 h exposure	[84]
	Yes			Increased cytotoxicity observed for methotrexate when delivered via PHBV magnetic nanoparticles	
PHB	Yes	HMEC	Cell viability	No cytotoxicity observed; cell viability remained high in blend films	[85]

**Table A2:** Studies included in the ecotoxicity data library for scl-PHA-based formulations data library.

Material Composition	Additive Presence	Test Organism	Endpoint	Observed Effect	Reference
PHB	No	Mouse	Acute oral toxicity	No acute toxicity	[86]
PHB	No	*Hydra Viridissima*	Population growth	Toxicity observed	[10]
PHBV	No	*Eisenia Andrei*	Reproduction	Reduced reproduction observed at high soil concentrations	[87]
PHB	Yes	*Sinapis Alba*	Seed Germination Index	No phytotoxicity observed following biodegradation	[88]
	No				
PHB	No	*Paracentrorous Lividus Larvae*	Toxic Units	Slightly Toxic	[89]
PHB	No	*Litopenaeus Vannamei*	Survival and growth	No acute toxicity; concentration-dependent reductions in survival and growth under long-term exposure	[90]
PHBV co 4HV 12% mol HV, 1% mol 4HV	No	*Artemia Franciscana*	Mortality	No acute toxicity observed following biodegradation	[91]
PHBV	No	*Zea Mays*	Plant Growth	Dose-dependent reduction in plant growth observed at low soil concentrations	[9]
PHB	No	*Danio Rerio*	Developmental Toxicity	Increased mortality and developmental abnormalities observed at tested concentrations	[92]
PHB	Yes	*Lactuca Sativa*, *Lycopersicon Esculentum*	Plant Growth	Growth inhibition observed following exposure to film extracts	[93]
PHB	Yes	*Lepidum Sativum*, *Hordeum Vulgare*	Plant Growth	No phytotoxicity observed in plant growth tests	[94]
	No				
PHBV 6.5% mol HV	No	*Aliivibrio Fischeri*, *Rhodomonas Salina*, *Paracentrotus Lividus*, *Mytilus Galloprovincialis*, *Acartia Tonsa*	Growth	Toxicity observed across multiple marine planktonic species; microalgae more sensitive	[95]
PHB	No	*Paracentrotus Lividus*	Developmental toxicity	Developmental delay and morphological abnormalities observed in embryos	[96]
PHB	No	*Idotea Balthica Basteri*	Mortality	Increased Mortality observed under chronic dietary exposure	[97]
PHB	No	*Lemna Minor*	Plant Growth	No adverse effects on plant growth observed	[98]
PHB	No	*Paracentrotus Lividus*	Developmental Growth	Developmental delay and morphological abnormalities observed in embryos	[99]
PHB	No	*Lactusa Sativa*, *Lycopersicon Esculentum*	Plant Growth	Severe inhibition of plant growth observed following exposure to mulch fragments	[100]
PHB	No	*Anabaena* Sp., *Chlamydomonas reinhardtii*, *Daphnia Magna*	Growth inhibition	Growth inhibition observed across multiple aquatic organisms following exposure to secondary PHB nanoplastics	[101]
PHB	No	*Daphnia Magna*	Behavior and feeding	Altered behavior and reduced feeding observed following exposure to PHB microplastics	[11]
PHB4V, 70% mol 4HV	No	*Rattus Norvegicus*	Acute/subacute oral toxicity	No mortality or adverse effects up to 2000 mg/kg	[102]

### Appendix A.2

Table A3 lists the 30 material features included in the PHB/PHBV cytotoxicity database, which is stored in the AUA Zenodo repository [28]. For each feature, the table shows the feature name as used in the dataset, a brief scientific description, the measurement unit (if applicable), and the observed value range across all samples. The materials properties dataset contains features describing material descriptors, including composition, additives, molecular descriptors, physicochemical properties, physical–morphological characteristics, and surface features of PHBV-based materials.

**Table A3:** Overview of all the descriptors of cytotoxicity of scl-PHA-based formulations data library.

Feature	Description	Units	Range	Type
Study_id	Unique identifier for study instance	None	varies	Categorical
Instance	Experimental run number	None	varies	Categorical
Monomer A	Primary monomer	None	varies	Categorical
Monomer B	Primary monomer	None	varies	Categorical
HB_ratio	Hydroxybutyrate ratio	mol%	0–100	Numerical
HV_ratio	Hydroxyvalerate ratio	mol%	0–100	Numerical
Molecular_weight (Mw)	Weight-average molar mass	g/moL	7300–6,800,000	Numerical
Molecular_Weight (Mn)	Number-average molar mass	kDa, g/moL	86–450,000	Numerical
Polydispersity_Index (PDI)	Polydispersity index	ratio	1–10	Numerical
Shape	Shape/morphology (film, particle, ribbon, etc.)	None	varies	Categorical
Dimensions	Dimensions of sample	mm, cm, µm	0.01–400	Numerical
Additives	Presence of incorporated additives	None	Yes/No	Categorical
Additive_name	Name of incorporated additives	None	varies	Categorical
Additive_Percentage	Additive content	%	0–100	Numerical
ISO	ISO standard used for cytotoxicity testing	None	varies	Categorical
ASTM	ASTM standard used for cytotoxicity testing	None	varies	Categorical
Assay	Type of cytotoxicity assay performed	None	varies	Categorical
Cell_Line	Name of the cell line used in the assay	None	varies	Categorical
Cell_Line_Type	Classification of the cell line (e.g., fibroblast, epithelial)	None	varies	Categorical
Exposure_Type	Method of sample exposure to cells	None	Direct/Indirect	Categorical
Number_of_Cells_per_well, Inoculum_concentration_units	Cell density used in each well of the assay plate	cells/well	2000–20,000	Numerical
Culture_Medium	Medium used to culture the cells	None	varies	Categorical
Dilution_Medium	Medium used for sample dilution	None	varies	Categorical
Concentration, Concentration_Units	Concentration of the test sample applied	mg/L, mg/mL, μg/mL, μΜ	0–2500	Numerical
Dilution	Dilution factor or ratio of the test sample before exposure	Ratio	0–1000	Numerical
Incubation_Time, Incubation_Time_Units	Duration of cell exposure to the test sample	hours	18–336	Numerical
Incubation_Temperature, Incubation_Temperature_Units	Temperature at which incubation was conducted	°C	37	Numerical
CO_2__Percentage	Percentage of CO_2_ used during incubation	%	5	Numerical
Air	Atmospheric conditions during incubation	None	Humidified	Categorical
Extraction_Time, Extraction_Time_Units	Duration of the extraction process	hours	24–72	Numerical
Extraction_ Temperature, Extraction_ Temperature Units	Temperature at which extraction was performed	°C	37	Numerical
Extraction_Medium	Medium used for extraction of leachables	None	varies	Categorical
Extraction_ Surface_Area, Extraction_ Surface_Area_Units	Ratio of material surface area to extraction medium volume	cm^2^/mL	0.5–1.25	Numerical
Number_of_cells_well	Number of viable cells remaining after exposure	cells/well	300–23,000	Numerical
Cell_viability_percentage	Percentage of viable cells after exposure	%	13–158	Numerical
Toxicity	Qualitative measure of cytotoxic effect based on a 70% cell viability threshold	None	Toxic/Nontoxic	Categorical
Growth_constant_μ, Growth_constant_μ_Units	Specific growth rate constant	h^−1^	0–2	Numerical
Relative_Growth_Rate	Growth rate relative to control	%	0–200	Numerical
Toxicity_Grade	Numerical classification of toxicity intensity (e.g., 0 = non-toxic, 4 = severe)	None	0–4	Numerical
Growth_Inhibition_Percentage	Percentage of growth inhibition relative to control	%	0–100	Numerical

Table A4 lists the 47 material features included in the PHB/PHBV ecotoxicity database, which is stored in the AUA Zenodo repository [29].

**Table A4:** Overview of all the descriptors of ecotoxicity of scl-PHA-based formulations data library.

Feature	Description	Units	Range	Type
Study_id	Unique identifier for study instance	None	varies	Categorical
Instance	Experimental run number	None	varies	Categorical
Monomer_A	Primary monomer	None	varies	Categorical
Monomer_B	Primary monomer	None	varies	Categorical
HB_ratio	Hydroxybutyrate ratio	mol%	0–100	Numerical
HV_ratio	Hydroxyvalerate ratio	mol%	0–100	Numerical
Molecular_Weight (Mw)	Weight-average molar mass	g/mol	163,000–350,200	Numerical
Molecular_Weight (Mn)	Number-average molar mass	g/mol	7800–300,000	Numerical
Polydispersity_Index (PDI)	Polydispersity index	ratio	1–10	Numerical
Shape	Shape/morphology (film, particle, ribbon, etc.)	None	Varies	Categorical
Dimensions	Dimensions of sample	mm, cm, µm	0.01–400	Numerical
Additives	Presence of incorporated additives	None	Limited	Categorical
Additive_Name	Name of incorporated additives	None	varies	Categorical
Additive_Percentage	Additive content	%	0–100	Numerical
Additive_Use	Type of additive (e.g., filler, plasticizer)	None	varies	Categorical
Polymer_Percentage	PHA content in final formulation	%	0–100	Numerical
Species_Name	Scientific name of the test organism	None	varies	Categorical
Species_Group	Taxonomic or functional group of the organism	None	Limited	Categorical
Initial_Organism_Age, Initial_Organism_Units	Age of the organism at the start of exposure	Days, weeks, months, g, μm	0–220	Numerical
Exposure_Type	Type of exposure applied during the test	None	Limited	Categorical
Media_Type	Type of test medium used (e.g., water, sediment, soil)	None	Limited	Categorical
Test_Location	Location or facility where the test was conducted	None	varies	Categorical
Tested_Concentration, Tested_Concentration_Units	Nominal or measured concentration of the tested substance	g/g, g/Kg, g/L, mg/Kg, mg/kg/day, mg/L, mL/L, μg/mL	0–2000	Numerical
Observation Period, Observation Period Units	Duration of the exposure or observation phase	Minutes, Hours, days, weeks	2–183	Numerical
Toxicity	Indicates whether the test represents acute or chronic toxicity	None	Limited	Categorical
EC10, EC10 Units	Concentration of the substance causing 10% effect on the test population	mg/L	0–1600	Numerical
EC50, EC50 Units	Concentration of the substance causing 50% effect on the test population	mg/L	0–4000	Numerical
Toxic_Units	Unit expressing toxicity level based on standard formula (e.g., 100/EC50)	None	0–14	Numerical
LC50, LC50_Units	Concentration of the substance lethal to 50% of the test organism	mg/L	0–100	Numerical
LD_50, LD_50 Units	Dose of the substance lethal to 50% of the test organisms	mg/kg	0–2000	Numerical
Mortality_Percentage	Percentage of organisms that died during the exposure period	%	0–100	Numerical
Body_Mass_Change_Percentage	Percentage of organisms that changed in body mass compared to control	%	0–100	Numerical
Size_Increase, Size_Increase Units	Change in organism size during exposure	g, μm	0–300	Numerical
Number_Of_ Juvenilles	Total number of juveniles produced per test unit	None	0–8	Numerical
Number_Of Cocoons	Total number of cocoons produced during the test	None	0–4	Numerical
SGI_Percentage	Seed Germination Index as a percentage of control	%	0–100	Numerical
Survival_Rate_Percentage	Percentage of organisms that survived exposure	%	0–100	Numerical
Normal_Plutei_Percentage	Percentage of organisms that survived exposure	%	0–100	Numerical
Specific_Growth_Rate, Specific_Growth_Rate_Units	Measure of how fast an organism (or population algae, fish, and daphnia) grows over time	%/day	0–100	Numerical
Weight_Gain, Weight Gain_Units	Increase in organism weight measured over time compared to control	%, g	0–200	Numerical
Plant_Height, Plant_Heights_Units	Average height of plants measured at test termination	cm	0–50	Numerical
Shoot_Biomass, Shoot_Biomass_Units	Dry or fresh weight of the shoot biomass	mg, g	0–80	Numerical
Foliar_C_N_ratio	Ratio of carbon to nitrogen in plant foliage	None	0–74	Numerical
Plant_Growth	Overall plant growth rate during exposure	%	0–100	Numerical
Toxicity_Grade	Numerical value indicating the severity or intensity of toxicity effects	None	0–2	Numerical
Toxicity_Classification	Classification of the substance’s toxicity according to GHS criteria	None	Limited	Categorical
